# Single-cell resolution long-term luciferase imaging in cultivated *Drosophila* brains

**DOI:** 10.17912/micropub.biology.000280

**Published:** 2020-07-22

**Authors:** Frank K. Schubert, Charlotte Helfrich-Förster, Dirk Rieger

**Affiliations:** 1 Neurobiology and Genetics, Biocenter, University of Würzburg, 97074 Würzburg

**Figure 1. Long-term cultivation and PER-LUC imaging of explanted Drosophila brains:**
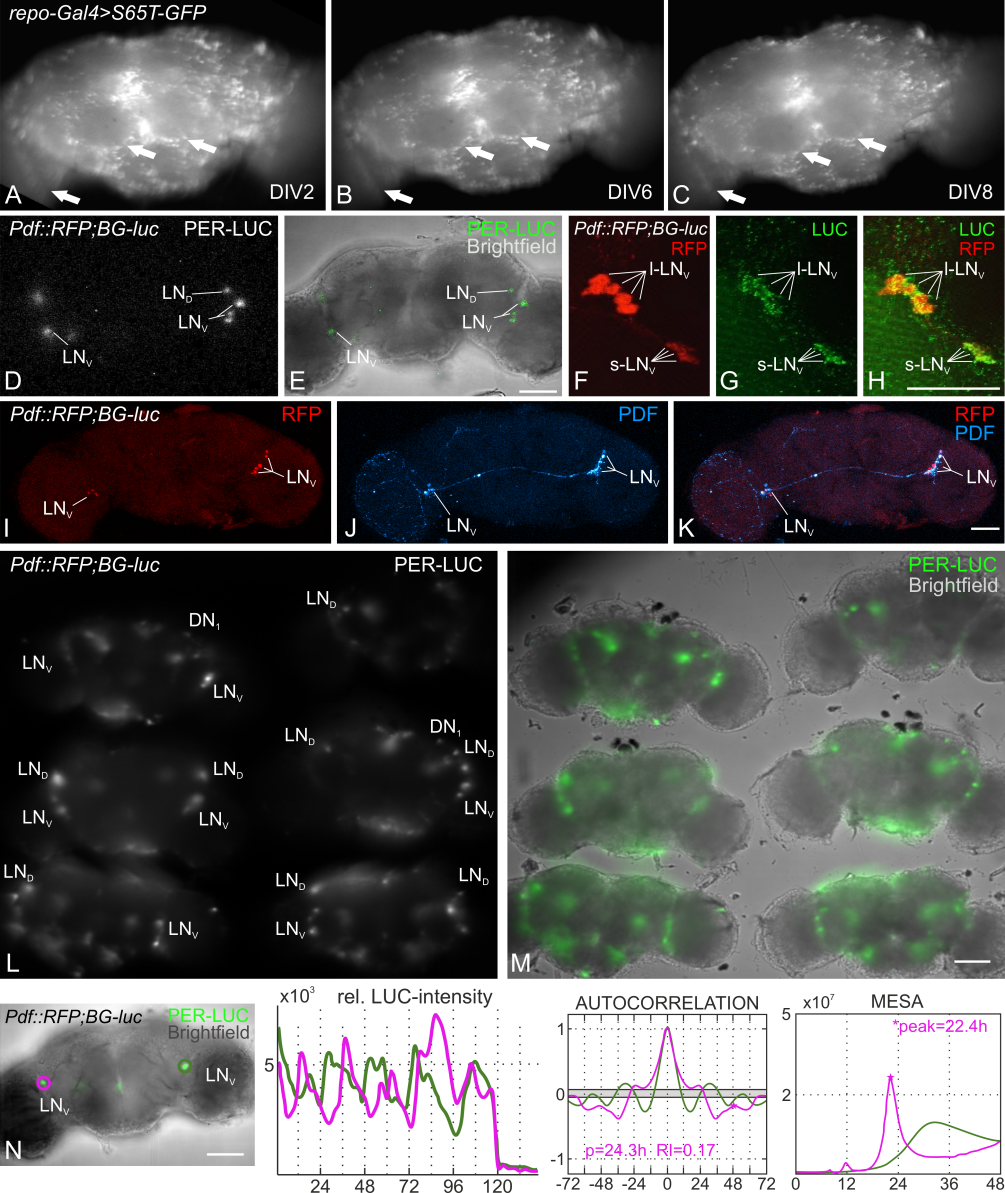
**A-C**: The viability of *repo-Gal4 > UAS-GFP-S65T* brain explants was monitored with a fluorescence stereomicroscope to determine how long the brain cultures could survive if the medium was not refreshed. Judging by eye, only a negligible number of GFP-expressing glial cells had vanished over the course of the first six days in culture, and prominent neuropil boundaries still looked defined (A, B; arrows). On the 7-9th day in vitro (DIV), the boundaries began to blur (medulla, antennal lobes; arrows), even though most glial cells still looked healthy (C). **D-E**: Single time-point of a PER-LUC bioluminescence measurement (D) and an overlay with the brightfield image of the brain from a test fly of the genotype *y w; Pdf::RFP1; BG-luc* (E). The relatively large size and the stereotypic localization in the anterior brain make the ventrolateral neurons (LNv) the easiest group to record from. **F-H**: Confocal scans of the native RFP expression (F) in the PDF expressing small and large ventrolateral neurons (s-LN_v_ and l-LN_v_, respectively) of the test flies (*y w; Pdf::RFP1; BG-luc*), counterstained with a Luciferase-antibody (LUC; G). **I-K**: Successfully imaged brains can be processed for immunohistochemistry. Native RFP expression in PDF neurons (I) and anti-PDF staining (J) of a brain, which had been kept in culture for seven days, prior to the immunohistochemistry procedure. The brain had been mounted in the culture dish 10 days post-dissection and was subsequently scanned with the 10-fold magnification air-objective of a confocal microscope. **L-M**: Average t-projected luminescence signal of a five-day long recording (measured every 10 minutes with 300 s exposure time per frame; L) and merge with the brightfield image (M). Up to six brains can be monitored simultaneously in one culture dish by using the 30-fold objective. **N**: Exemplary brain and analysis of the measured PER-LUC oscillations of lateral neurons. For correct assignment of the plots, a color code was used to highlight the recorded cells (green and magenta circles). The graph next to the recorded brains shows the lowpass filtered and background subtracted bioluminescence intensity over time (in hours). The plot in the center consists of the respective correlograms of the autocorrelation analysis. A solid gray bar indicates the 5% significance level. Detected rhythms were further analyzed for period length (p) and rhythmicity index (RI). The results of the MESA are shown in the rightmost plot. The peaks of the correlograms indicate the estimated period lengths (x-axis). Scale bars = 50 μm. LN_v_: ventrolateral clock neurons, s-LN_v_: small ventrolateral clock neurons, l-LN_v_: large ventrolateral clock neurons, LN_D_: dorsolateral clock neurons, DN_1_: dorsal clock neurons, PDF: pigment dispersing factor.

## Description

In *Drosophila melanogaster*, approximately 150 so-called clock neurons express the molecular components of the circadian clock mechanism. The activity of these neurons enables the fly to anticipate and track cyclic changes in their environment and to respond to them accordingly. The core of the circadian clock is built by two interlocked transcriptional feedback loops. In the first loop, the transcription factors CLOCK (CLK) and CYCLE (CYC) initiate the expression of the core clock proteins PERIOD (PER) and TIMELESS (TIM), which are in turn repressing the activity of CLK and CYC as transcriptional activators, resulting in a cyclic accumulation of PER and TIM (reviewed in Hermann-Luibl and Helfrich-Förster, 2015). In light:dark cycles, the intracellular photoreceptor Cryptochrome (CRY) mediates light information to the core clock and resets the molecular mechanism, thereby synchronizing the clock every day at sunrise to the 24h oscillation (reviewed by Helfrich-Förster, 2020). However, in constant darkness, the oscillation of the clock proteins PER and TIM cycles with the endogenous period, which is close to, but not exactly 24hrs.

A commonly used read-out for the molecular oscillations are immunohistochemical assays to quantify the clock protein abundance over time. For this endeavor, the researcher has to dissect and analyze a large number of animals, which is very labor-intensive and time-consuming. A standard timeseries in *Drosophila* chronobiology takes at least 80 flies per genotype, when the sampling occurs every 3hrs (8 timepoints, 10 flies per timepoint).

In 2011, the lab of Herman Wijnen was the first to describe a procedure which enables to assess the clock protein cycling of single clock neurons in individual brains of *Drosophila* by imaging PER-Luciferase (PER-LUC) activity (Sellix *et al.*, 2010). Even though this technique has the potential to overcome the labour-intensive timeseries sampling for immunohistochemical experiments, only one more study utilized the single-cell luciferase imaging approach resulting in two publications (Roberts *et al.*, 2015 and Roberts *et al.*, 2016). We believe that this is on one hand partially caused by the complicated cultivation protocols available and on the other hand that the two mentioned studies have been performed on custom-built microscope setups, which might deter potential users. (Author comment: An additional study using a similar experimental setup as described in this article has been uploaded to the bioRxiv preprint server while this article was under review (Versteven *et al.*, 2020)).

In this article, we briefly describe a culturing protocol for explanted *Drosophila* brains, which is sufficient to keep the brains alive and healthy for more than a week, even without partial substitution of the culture medium on a regular basis. We further used a commercial Olympus Luminoview (LV200) luminescence microscope equipped with an EMCCD camera (ImagEm X2 9100-23B, Hamamatsu Photonics) to monitor the PER-LUC expression of single clock neurons over several circadian cycles to show the viability of the explanted brains in an independent assay.

The flies for this experiment were reared under 12:12 hr light:dark-cycles on standard corn agar medium at 25°C with 60% relative air humidity. After dissection in ice cold Ca^2+^ free Ringer’s solution (see reagents), we placed the brains in a Poly-L-Lysine coated glass bottom petri dish (Greiner Bio-One) and added the culture medium. The culture medium contains 20% heat-inactivated fetal bovine serum, 1% Penicillin-Streptomycin and 0.75 µM Luciferin. Subsequently, the dish with the brain explants was transferred either to a culture incubator or to the luminescence microscope for imaging. For avoiding movement artifacts during long-term recording, we decided to culture the brain explants without partially substituting the culturing medium. To test the viability of these explants, we assessed pan-glial EGFP expression over the course of several days, since glia are essential for neuronal survival and suppression of cell death (Buchanan and Benzer, 1993; Xiong and Montell, 1995; Volkenhoff *et al.*, 2015). We could not observe any dramatic loss of glial cells after eight days in vitro (DIV8) without adding fresh medium (Fig. 1 A-C). The majority of the targeted cells express EGFP on DIV8, and we therefore concluded that the explants are viable for at least eight days in culture. In the next step we assayed brains from the *y w; Pdf::RFP1; BG-luc* line (expressing PER-LUC in all clock neurons and RFP under the control of the *Pdf* promotor) under the LV200 luminescence microscope. The resolution of the setup is sufficient to identify single clock neurons by their PER-LUC expression by using either 30-fold or 60-fold super apochromat objectives and 300 s exposure time (UPLSAPO 30x, UPLSAPO 60x, Olympus life science; Fig. 1 D, and 1 L, respectively). The specificity of the *y w; Pdf::RFP1; BG-luc* line was confirmed by analyzing the native *Pdf*::mRFP1 expression and a separate staining against the firefly luciferase, which was colocalized in the Pigment Dispersing Factor expressing (PDF^+^) ventrolateral clock neurons (Fig. 1 F-H). We could further show that the staining for the circadian output factor PDF also worked after the brain had been imaged and kept in culture for eight consecutive days, showing that the tissue is indeed viable throughout the complete recording time (Fig. 1 I-K). Even though it was not possible to record z-stacks of the cultivated brains with the LV200 due to the long exposure time (300 s for one plane), the simultaneous recording of up to six synchronized brains is an option to increase the probability of recording several circadian clock clusters of different focal planes and/ or to increase sample size (Fig. 1 L, M).

The analysis of the recorded large ventrolateral clock neurons’ (l-LNv) PER-LUC rhythms via a MATLAB toolbox (Levine *et al.*, 2002) identified a protein cycling with a circadian period of approximately 24hrs in one of the neurons (magenta circled neuron, Fig. 1 N). The period length estimated by MESA is shorter than the one obtained with autocorrelation analysis (22.4hrs and 24.3hrs, respectively; Fig. 1 N). In the other neuron both methods failed to detect rhythmicity, even though the oscillations appeared rhythmic (green circled neuron, Fig. 1 N). We estimated the period length by manually measuring the peak-to-peak intervals. Although the underlying rhythm is not significant, the manually calculated period length of approximately 23.6hrs falls into the circadian range. It is reported that the l-LNv would not cycle in constant darkness in the intact fly (Grima *et al.*, 2004), but the aforementioned studies observe a PER cycling in those cells as well (Sellix *et al.*, 2010, Roberts *et al.*, 2015, Roberts *et al.*, 2016). This is most likely due to the loss of inhibitory signals from the periphery (e.g. photoreceptors, Schlichting *et al.*, 2016).

We therefore conclude that, depending on the study aim, the recording of luciferase reporter expression of explanted brains is a well-suited alternative to the classical work-intensive immunohistochemical time-course experiments and combines a higher resolution of the clock protein cycling together with a smaller number of animals that need to be studied.

## Reagents

**Fly lines:**

*y w; Pdf::RFP1; BG-luc* (y w; P{Pdf-RFP.R}; P{BG-luc}); expresses mRFP1 under the control of the *Pdf* promotor and a PERIOD-LUCIFERASE fusion protein in all clock neurons. See Ruben *et al.* (2012) for information on the *Pdf::mRFP1* construct and Stanewsky *et al.* (1997) for information on the *BG-luc* line.

*Repo-Gal4* (w^1118^; P{w^+m*^=GAL4}repo/TM3,Sb^1^; Bloomington stock #7415); expresses GAL4 in glia cells. See Sepp *et al.* (2001) for further information.

*UAS-S65T-GFP* (w^*^; P{w^+mC^=UAS-GFP.S65T}eg^T10^; Bloomington stock #1522); expresses GFP.S65T under the control of UAS.

**Primary antibodies:**

Anti-LUC (Thermo Fisher Scientific, RRID: AB_1076533); raised in mouse, binds to the Luciferase (LUC) enzyme. Used in a final concentration of 1:2000.

Anti-mCherry (Thermo Fisher Scientific, RRID: AB_2536611); raised in rat, binds to red fluorescent protein (RFP) and its derivates. Used in a final concentration of 1:2000.

Anti-PDF-C7 (DSHB, deposited by J. Blau in 2005, RRID: AB_760350); raised in mouse, binds to the pigment dispersing factor (PDF) neuropeptide expressed by the lateral clock neurons. Used in a final concentration of 1:4000.

**Secondary antibodies:**

AlexaFluor488 anti mouse (Thermo Fisher Scientific, RRID: AB_2534069); used in a final concentration of 1:200.

AlexaFluor568 anti rat (Thermo Fisher Scientific, RRID: AB_2534121); used in a final concentration of 1:200.

AlexaFluor647 anti mouse(Thermo Fisher Scientific, RRID: AB_2535804); used in a final concentration of 1:200.

**Buffers and solutions:**

Ca^2+^ free Ringer’s solution (70 mM NaCl, 5 mM KCl, 20 mM MgCl_2_, 10 mM NaHCO_3_, 120mM Sucrose, 5mM HEPES; recipe shared by André Klarsfeld and Serge Birman)

Culture medium (SM with 20% FBS, 1% Pen/Strep and 0.75µM Luciferin; recipe shared by André Klarsfeld and Serge Birman)

Fetal bovine serum (FBS, PAA #A15-101)

Luciferin (Biosynth #L-8220)

Paraformaldehyde 4% (PFA, Affymetrix #199431LT)

Penicillin/ Streptomycin (Pen/Strep, PAA #P11-010)

Phosphate buffered Saline (PBS, Sigma-Aldrich #P5493)

Poly-L-lysine (Sigma-Aldrich #P4707)

Schneider’s insect medium (SM, Sigma-Aldrich #S0146)

Glass bottom culture dish Greiner Bio-One #627861

## References

[R1] Buchanan RL, Benzer S (1993). Defective glia in the Drosophila brain degeneration mutant drop-dead.. Neuron.

[R2] Grima B, Chélot E, Xia R, Rouyer F (2004). Morning and evening peaks of activity rely on different clock neurons of the Drosophila brain.. Nature.

[R3] Helfrich-Förster C (2019). Light input pathways to the circadian clock of insects with an emphasis on the fruit fly Drosophila melanogaster.. J Comp Physiol A Neuroethol Sens Neural Behav Physiol.

[R4] Hermann-Luibl C. and Helfrich-Förster C. (2015). Clock network in <i>Drosophila. Curr Opin Insect Sci</i> 7:65-70. 10.1016/j.cois.2014.11.003 32846682

[R5] Levine JD, Funes P, Dowse HB, Hall JC (2002). Advanced analysis of a cryptochrome mutation's effects on the robustness and phase of molecular cycles in isolated peripheral tissues of Drosophila.. BMC Neurosci.

[R6] Roberts L, Leise TL, Noguchi T, Galschiodt AM, Houl JH, Welsh DK, Holmes TC (2015). Light evokes rapid circadian network oscillator desynchrony followed by gradual phase retuning of synchrony.. Curr Biol.

[R7] Roberts L, Leise TL, Welsh DK, Holmes TC (2016). Functional Contributions of Strong and Weak Cellular Oscillators to Synchrony and Light-shifted Phase Dynamics.. J Biol Rhythms.

[R8] Ruben M, Drapeau MD, Mizrak D, Blau J (2012). A mechanism for circadian control of pacemaker neuron excitability.. J Biol Rhythms.

[R9] Schlichting M, Menegazzi P, Lelito KR, Yao Z, Buhl E, Dalla Benetta E, Bahle A, Denike J, Hodge JJ, Helfrich-Förster C, Shafer OT (2016). A Neural Network Underlying Circadian Entrainment and Photoperiodic Adjustment of Sleep and Activity in Drosophila.. J Neurosci.

[R10] Sellix MT, Currie J, Menaker M, Wijnen H (2010). Fluorescence/luminescence circadian imaging of complex tissues at single-cell resolution.. J Biol Rhythms.

[R11] Sepp KJ, Schulte J, Auld VJ (2001). Peripheral glia direct axon guidance across the CNS/PNS transition zone.. Dev Biol.

[R12] Stanewsky R, Jamison CF, Plautz JD, Kay SA, Hall JC (1997). Multiple circadian-regulated elements contribute to cycling period gene expression in Drosophila.. EMBO J.

[R13] Versteven M., Ernst K-M., and Stanewsky R. (2020). A robust self-sustained peripheral circadian oscillator reveals differences in temperature compensation properties with central brain clocks. <i>bioRxiv</i> 2020.06.24.168450.This article is a preprint and has not been certified by peer review 10.1016/j.isci.2020.101388 PMC7452380 32798967

[R14] Volkenhoff A, Weiler A, Letzel M, Stehling M, Klämbt C, Schirmeier S (2015). Glial Glycolysis Is Essential for Neuronal Survival in Drosophila.. Cell Metab.

[R15] Xiong WC, Montell C (1995). Defective glia induce neuronal apoptosis in the repo visual system of Drosophila.. Neuron.

